# Changes in haemolymph parameters and insect ability to respond to immune challenge during overwintering

**DOI:** 10.1002/ece3.7323

**Published:** 2021-03-11

**Authors:** Michal Řeřicha, Pavel Dobeš, Michal Knapp

**Affiliations:** ^1^ Department of Ecology Faculty of Environmental Sciences Czech University of Life Sciences Prague Prague ‐ Suchdol Czech Republic; ^2^ Department of Experimental Biology Faculty of Science Masaryk University Brno Czech Republic

**Keywords:** antimicrobial response, bacterial challenge, Climate change, cold tolerance, fluctuating temperatures, haemocytes, innate immunity, invasive species, trade‐off

## Abstract

Overwintering is a challenging period in the life of temperate insects. A limited energy budget characteristic of this period can result in reduced investment in immune system. Here, we investigated selected physiological and immunological parameters in laboratory‐reared and field‐collected harlequin ladybirds (*Harmonia axyridis*). For laboratory‐reared beetles, we focused on the effects of winter temperature regime (cold, average, or warm winter) on total haemocyte concentration aiming to investigate potential effects of ongoing climate change on immune system in overwintering insects. We recorded strong reduction in haemocyte concentration during winter; however, there were only limited effects of winter temperature regime on changes in haemocyte concentration in the course of overwintering. For field‐collected beetles, we measured additional parameters, specifically: total protein concentration, antimicrobial activity against *Escherichia coli,* and haemocyte concentration before and after overwintering. The field experiment did not investigate effects of winter temperature, but focused on changes in inducibility of insect immune system during overwintering, that is, measured parameters were compared between naïve beetles and those challenged by *Escherichia coli*. Haemocyte concentration decreased during overwintering, but only in individuals challenged by *Escherichia coli*. Prior to overwintering, the challenged beetles had a significantly higher haemocyte concentration compared to naïve beetles, whereas no difference was observed after overwintering. A similar pattern was observed also for antimicrobial activity against *Escherichia coli* as challenged beetles outperformed naïve beetles before overwintering, but not after winter. In both sexes, total protein concentration increased in the course of overwintering, but females had a significantly higher total protein concentration in their hemolymph compared to males. In general, our results revealed that insect’s ability to respond to an immune challenge is significantly reduced in the course of overwintering.

## Graphical abstract

Prior to overwintering, the challenged insects had a significantly higher haemocyte concentration compared to naïve insects, whereas no difference was observed after overwintering. A similar pattern was observed also for antimicrobial activity against *Escherichia coli* as challenged insects outperformed naïve insects before overwintering, but not after winter.

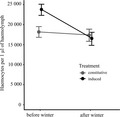

## 1 INTRODUCTION

Winter period is energetically challenging for temperate insects, as food resources are very limited and great majority of species are not feeding at all in the course of overwintering (Lee et al. 1991). In addition to basic body maintenance, some energy is needed also for improvement of cold hardiness (e.g., production of cryoprotectants; Watanabe 2002). A great majority of temperate insects minimize energy expenditures during overwintering by entering the dormant state (diapause, quiescence; Hahn and Denlinger 2007, 2011, Williams et al. 2015). As the immune system of insects is energetically costly to maintain, a substantial investment into the immune system can compromise insect survival during winter (Ferguson et al. 2018a). Thus insects face a trade‐off between investment into the immune system and energy storage in the cold. These responses can be species‐specific, depending on species traits and environmental conditions experienced (Ferguson et al. 2016, 2018a). Moreover, the lowered activity of immune system during overwintering can be a result of both slow biochemical reactions at low temperatures and evolutionary adaptation to limited pathogen pressure during winter (Ferguson and Sinclair 2017, Ferguson et al. 2018a).

Winter temperatures can strongly affect the overwintering success of insects (Bale and Hayward 2010, Williams et al. 2015, Ferguson et al. 2016, Ferguson and Sinclair 2017). Interestingly, the majority of “climate change” research have explored the effects of temperature on insects during growing season, whereas far less is known about the effects of temperatures during the winter period (Bale and Hayward 2010, Williams et al. 2015), and only a small part of these studies focused on effects of winter temperature on insect immune system (Vesterlund et al. 2014, Urbański et al. 2017, Ferguson et al. 2018b, Körner et al. 2018). Overwintering insects have to overcome hostile environmental conditions, for example, food and water deficit and low temperatures causing chill injuries (Williams et al. 2015, Ferguson et al. 2018a). The winter temperature does not only affect insects but also activity and impact of their pathogens and thus corresponding changes in insect immune system are expected (Ferguson et al. 2018a).

The innate immune system of insects includes a variety of cellular and humoral components that together maintain homeostasis of individual organisms, that is, ensure the removal of pathogens as well as damaged tissues. Typical cellular reactions in insects are mediated by immune cells called haemocytes and include phagocytosis, encapsulation, and nodulation (Stanley and Miller 2006, Strand 2008). Humoral immunity involves, for example, the activity of phenoloxidase cascade, lectins, lysins, and specific antimicrobial peptides (AMPs; Rosales 2018, Sheehan et al. 2018). Low temperatures experienced during overwintering have been shown to lead, for instance, to increased risk of fungal infection, caused by genera *Beauveria* or *Metarhizium* (Fernandes et al. 2008). In insects, the optimal temperature for haemocyte‐mediated immune responses is frequently lower than the optimal temperature of other biochemical reactions (Ferguson et al. 2018a). However, whether cellular or humoral components are more seriously affected by exposure to cold is not known and responses seem to be species‐specific (Nakamura et al. 2011, Ferguson et al. 2016, 2018b, Ferguson and Sinclair 2017, Urbański et al. 2017). In general, winter season is often immunosuppressive for temperate insect, and thermal performance of the immune system is believed to be optimized for ambient temperatures experienced by particular insect species (Ferguson et al. 2018a). For example, Nakamura et al. (2011) showed that exposition to low temperatures under laboratory conditions reduced the phagocytic ability of haemocytes in butterfly *Samia cynthia pryeri* and decrease in the total haemocyte count was recorded for overwintering carrion beetle *Nicrophorus vespilloides* (Urbański et al. 2017).

In this study, we investigated the effects of overwintering on physiological and immune parameters in the laboratory‐reared and field‐collected invasive harlequin ladybirds (*Harmonia axyridis* (Pallas, 1773)). For laboratory‐reared beetles we tested the effects of three temperature regimes corresponding to cold, average and warm winter and measured haemocyte concentrations during three sampling periods (before winter, peak winter, and after overwintering). We predicted that lower winter temperatures will result in decreased haemocyte concentrations due to slower haemocyte production resulting from slower biochemical reactions at low temperatures (Ferguson et al. 2018a). However, extremely cold winters can also lead to chill injuries, and this can be perceived as a challenge for the immune system and can enhance its activity (Chen et al. 1987, Irwin et al. 2003). As we did not observe strong effects of winter temperature on haemocyte concentration in our laboratory experiment, we decided to focus on inducibility of *H. axyridis* immune system in the following field experiment. For field‐collected beetles, we compared measurements of total haemocyte concentration, total protein concentrations, and antimicrobial activity against *Escherichia coli* between individuals sampled before and after overwintering periods. All parameters were measured for naïve as well as for *E. coli* challenged beetles. We hypothesized that a long period of low temperatures during overwintering and depleted energy reserves will result in ladybird inability to respond to immune challenge efficiently with the advancing winter season.

## 2 MATERIALS AND METHODS

### 2.1 Study species

The harlequin ladybird, *H. axyridis*, is one of the most invasive insect species in the world (Brown et al. 2011, Lombaert et al. 2011). Its native range is situated in East Asia, but the species has recently invaded Europe, North and South America, Africa, and West Asia (Roy et al. 2016). As typical of ladybirds, *H. axyridis* overwinters as an adult (Danks 1987, Hodek et al. 2012). On sunny autumn days, mass flights to overwintering sites can be observed (Obata 1986) and winter aggregations (up to thousands of individuals) are established in sheltered places that buffer against low winter temperatures (e.g., window frames, old buildings, or caves; Labrie et al. 2008). The adults have only a weak winter diapause, commonly terminated in January (peak winter in temperate climate) and followed by a quiescence phase (Raak‐van den Berg et al. 2012). *H. axyridis* has an extremely powerful immune system which has been proposed to be partly responsible for its successful invasion (Verheggen et al. 2017). The hemolymph of *H. axyridis* shows significantly higher antimicrobial activity than the hemolymph of some native European ladybirds, for example, *Coccinella septempunctata* (Gross et al. 2010, Knapp et al. 2018). The possession of the specific alkaloid harmonine could be partly responsible for this difference (Schmidtberg et al. 2013).

### 2.2 Experimental animals

In our experiments, we used two groups of beetles: laboratory‐reared beetles (in 2015; Experiment 1) and field‐collected beetles (in 2016; Experiment 2). The parents of laboratory‐reared beetles were collected in August 2015, in the campus of the Czech University of Life Sciences Prague, Czech Republic (GPS: 50°8′ N, 14°21′ E; 300 m a.s.l.). Parental pairs were formed at random and the next generation was bred from eggs laid by mated females (for details see: Knapp and Řeřicha 2020). Newly hatched larvae were fed ad libitum with eggs of *Ephestia kuehniella* (Zeller, 1879) (Lepidoptera: Pyralidae) and provided water in cotton wool. Adult laboratory‐reared beetles (emerged between September 15 and 25) were sexed, placed individually into Petri dishes, provided food and water ad libitum and exposed to the same standardized conditions as larvae until October 7, 2015, when the initiation of winter diapause was started. Laboratory‐reared ladybirds were used for the experiment investigating the effects of overwintering stage and winter temperature on total concentration of circulating haemocytes.

Field‐collected ladybirds were sampled in autumn 2016 (mid‐October) and were immediately used for the experiment investigating the effects of overwintering and immune challenge on basic immune and physiological parameters. Beetles were kept under outdoor conditions in large groups (ca 50 individuals) in glass jars of 1 liter with perforated lids to match microclimatic conditions within jars with ambient environment. Piece of crumpled paper was inserted to each jar to provide ladybirds with a suitable overwintering substrate. Jars were placed on the soil surface under a vegetation buffering jars from extreme temperatures. In Experiment 2, we investigated individuals from three geographically distant *H. axyridis* populations originating from Konětopy (GPS: 50°3′ N, 14°65′ E; 180 m a.s.l.), Malá Amerika (GPS: 49°95′ N, 14°18′ E; 380 m a.s.l.) and Hostim (GPS: 49°96′ N, 14°13′ E; 260 m a.s.l.). The individuals originating from Konětopy were relocated into four different microhabitats with specific temperature and humidity. The individuals from Malá Amerika and Hostim were exposed to environmental conditions natural to these sites (see temperatures recorded using dataloggers placed at overwintering sites, for details; Supporting Information Table S1).

### 2.3 Experiment 1 (laboratory‐reared beetles)

The overwintering part of Experiment 1 started on October 15, 2015. The beetles were exposed to three winter temperature regimes: 1) warm winter, 2) average winter and 3) cold winter, using computer‐controlled climatic chambers (see Knapp and Řeřicha 2020, for details). The temperature was adjusted every hour with a new temperature to mimic real temperatures experienced by *H. axyridis* in outdoor shelters in Central Europe (the temperatures applied are attached as Supporting Information file Table S2). The mean temperature (November to March) for the warm, average, and cold regimes were 2.8 °C, 0.7 °C, and ‐1.6 °C, respectively (the corresponding minimum temperature reached were ‐2.0 °C, ‐4.1 °C and ‐8.1 °C, respectively). Employed winter temperature regimes were fully within ecologically relevant temperatures experienced by *H. axyridis* and species ecophysiological limits (supercooling point is ca. ‐17 °C in *H. axyridis*; Berkvens et al. 2010). The ladybirds were overwintered in complete darkness allowing to maintain their diapause stage during the laboratory experiment.

The hemolymph was collected with a glass microcapillary tube (Hirschmann, Germany), using the reflex bleeding or the puncture method. During the reflex bleeding method, ladybirds were stimulated by poking their legs with an entomological pin to induce reflex bleeding behavior, after which a sample of reflex blood was collected. Puncture method consists of puncturing the metasternum of the ladybird with a sterilized entomological pin (diameter of 0.30 mm) and collecting the hemolymph that exuded from the puncture. Puncture method was used in the case that the reflex bleeding method failed (Knapp et al., 2018). The hemolymph samples were collected in three periods: before overwintering (October 15, 2015), in peak winter (January 20, 2016) and after overwintering (March 15, 2016). Sampled beetles were selected randomly from live individuals of each experimental temperature regime. Each individual was sampled only once and was then removed from the experiment after its sampling (n = 255). Note that high winter mortality of laboratory‐reared beetles resulted in a limited number of individuals sampled in the last period, that is, after overwintering, especially for the cold winter treatment (n_warm_ = 36, n_average_ = 33, n_cold_ = 12; see our dataset in Supporting Information Table S3). In our previous study (Knapp and Řeřicha 2020), we showed that winter survival was significantly lower for laboratory‐reared compare to field‐collected ladybirds. We suppose that environmental stability experienced during ladybird development or food quality, for example, *Ephestia* egg diet can limit ladybird capacity to produce cryoprotectants, may be responsible for this pattern.

### 2.4 Experiment 2 (field‐collected beetles)

Field‐collected beetles were transported to the laboratory in two groups, before their overwintering in mid‐October 2016 (n = 81) for the first time, and after overwintering in mid‐March 2017 (n = 156) for the second time. Before the hemolymph sampling, ladybirds were individually accommodated in Petri dishes (9 cm in diameter) and all beetles were transferred to computer‐controlled climatic chambers, set to a LD 16:8 h photoperiod at 23°C and 70% relative humidity for 48 h, that is, until the hemolymph sampling. We measured constitutive (baseline) immunity as well as induced immune activity (response to bacterial challenge). To measure the induced immune activity, we pricked half of the experimental animals by entomological pin dipped in bacterial suspension containing high concentration of live Gram‐negative bacteria *Escherichia coli* (CCM 3954) after 24 hours in the climatic chamber. Induced immune activity thus represented response to infection (after 24 hours). The hemolymph was collected in the same way as from laboratory‐reared beetles (see Knapp et al. 2018, for details). Each beetle was sampled only once and was then removed from the experiment.

### 2.5 Determination of physiological and immune parameters

The collected hemolymph was immediately diluted (100 × dilution) in an anticoagulant buffer (62 mM NaCl, 100 mM glucose, 30 mM trisodium citrate, and 26 mM citric acid, 10mM ethylenediaminetetraacetic acid; Firlej et al. 2012). In both experiments (Experiment 1 and 2), the haemocyte concentration of all investigated beetles was counted immediately after hemolymph collection using a Bürker chamber under a CX31 microscope (Olympus, Japan) with a magnification of 400x. The hemolymph samples collected from field‐collected beetles (Experiment 2) were stored in a freezer (at ‐20°C) and used for subsequent measurements of total protein concentration and antimicrobial activity against *E. coli*. Protein concentration in the hemolymph was measured colorimetrically by a Bradford protein assay (for details see Řeřicha et al. 2018). Antimicrobial activity against gram‐negative bacteria *E. coli* K12 was measured luminometrically using bioluminescent bacteria containing luxABCDEamp plasmid producing bacterial luciferase (Vojtek et al. 2014). The luminescence signal was recorded using a Chameleon V luminometer (Hidex, Finland) in counts per second. The antimicrobial activity was expressed proportional bacterial mortality during 30 minutes after exposition to diluted ladybird hemolymph mixed with bacterial working solution (for details see Vojtek et al. 2014; Řeřicha et al. 2018).

### 2.6 Data analyses

The first dataset, based on data for laboratory‐reared beetles (Experiment 1), is designed to investigate the effects of three winter temperature regimes (warm, average, and cold) on changes in haemocyte concentration in the course of overwintering. Raw data serving as input for the respective analyses are available in the Supporting Information file (Table S3). The second dataset, based on data for field‐collected beetles (Experiment 2), is designed to investigate effects of overwintering and immune challenge on selected hemolymph parameters. Raw data serving as input for the respective analyses are available in the Supporting Information file (Table S4). Data on haemocyte and protein concentrations were logarithmically transformed prior to analyses. Data on antimicrobial activity against *E*. *coli* were arcsine transformed prior to analysis.

**FIGURE 1:**
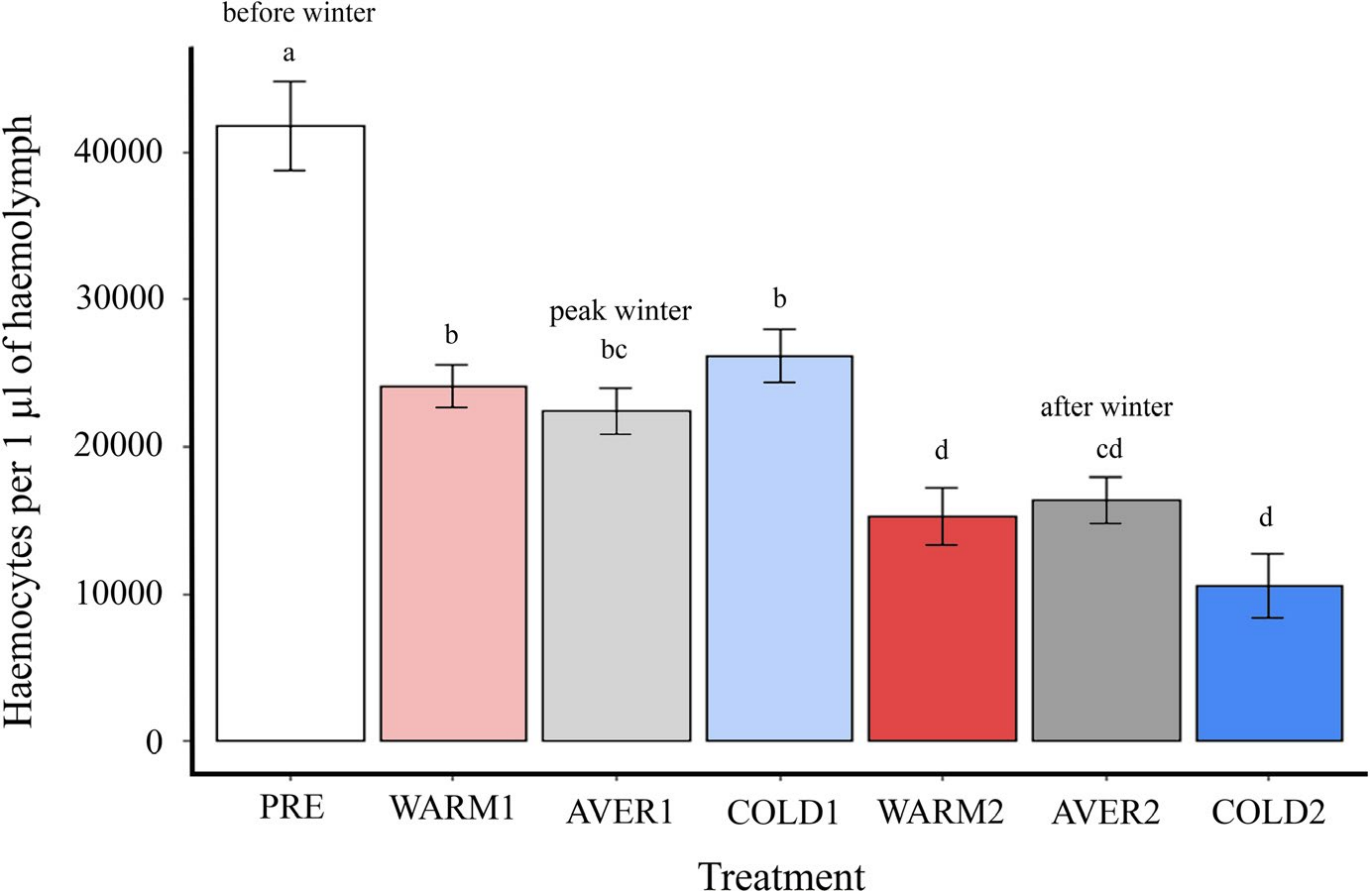
Effects of overwintering phase and temperature regime on haemocyte concentrations in the laboratory‐reared *Harmonia axyridis*. PRE = individuals sampled before winter; WARM = individuals kept under warm winter temperature conditions, AVER = individuals kept under average winter temperature conditions, COLD = individuals kept under cold winter temperature conditions. Symbol “1” indicates peak winter period and “2” indicates the end of overwintering. Means ± SEM are shown for each treatment and significant differences between treatments are indicated by different letters (Tukey’s post hoc test: P < 0.05)

Linear models (LMs) were applied to analyze the effects of overwintering and winter temperature on haemocyte concentration in laboratory‐reared ladybirds. The model to distinguish between the effects of sampling period during winter (peak winter or after overwintering) and winter temperature (Model 1) included sex, sampling period (peak winter or after overwintering), winter temperature regime (warm, average, or cold), and all possible interactions between the main terms as independent variables. The model to compare haemocyte concentration levels among all treatments (Model 2), included sex and “treatment,” that is, the latter being a factorial variable with seven levels representing all available combinations of sampling period and winter temperature (see Figure 1), as the only independent variables. This model was fitted to allow comparison of pairwise differences between all treatments (including preoverwintering treatment) using Tukey’s post hoc tests.

Linear mixed models (LMEs) were applied to analyze the effects of overwintering and immune challenge on selected hemolymph parameters in field‐collected beetles. Separate models were performed for haemocyte concentration, total protein concentration and antimicrobial activity against *E. coli*. Site identity (site where beetles spent winter; six sites in total) was used as a random effect in all analyses. The models included sex, sampling time (autumn/spring), treatment (constitutive/ induced immunity), and all possible interactions between the main terms as independent variables.

As the last step, we explored correlations between investigated parameters (total protein concentration, antimicrobial activity against *E. coli,* and haemocyte concentration) at the individual level using Spearman correlation tests. All data analyses were performed in R version 3.6.1 (R Development Core Team 2019), and LMEs were fitted using the “lme” function implemented in the “nlme” package (Pinheiro et al. 2020).

## 3 RESULTS

### 3.1 Effect of overwintering phase and temperature on haemocyte concentration in laboratory‐reared beetles

The concentration of circulating haemocytes differed significantly between treatments (LM – model 2: F_6,247_ = 49.68, P < 0.001; Figure 1), changing significantly from peak winter to the end of overwintering period (LM – model 1: F_1,195_ = 58.57, P < 0.001). Surprisingly, there was no direct significant effect of winter temperature on haemocyte concentration (LM – model 1: F_2,195_ = 0.12, P = 0.89; see also post hoc test results included in Figure 1). Haemocyte concentration did not differ between females and males (LM – model 1: F_1,195_ = 3.20, P = 0.075); however, there was a marginally significant interaction between sex and winter temperature (LM – model 1: F_2,195_ = 3.16, P = 0.045). Haemocyte decrease was less steep in warm and average temperature regimes compared to cold regime in males but not in females. Interestingly, there was a significant interaction between sampling period and temperature (LM – model 1: F_2,195_ = 6.14, P = 0.003), indicating that haemocyte concentration decreased less steeply toward the end of overwintering period at average temperature regime compared to cold temperature regime (Figure 1). Other interactions between the main terms were not significant (LM – model 1: both F < 0.67, both P > 0.41).

### 3.2 Effect of overwintering and immune challenge on hemolymph parameters in field‐collected beetles

**FIGURE 2:**
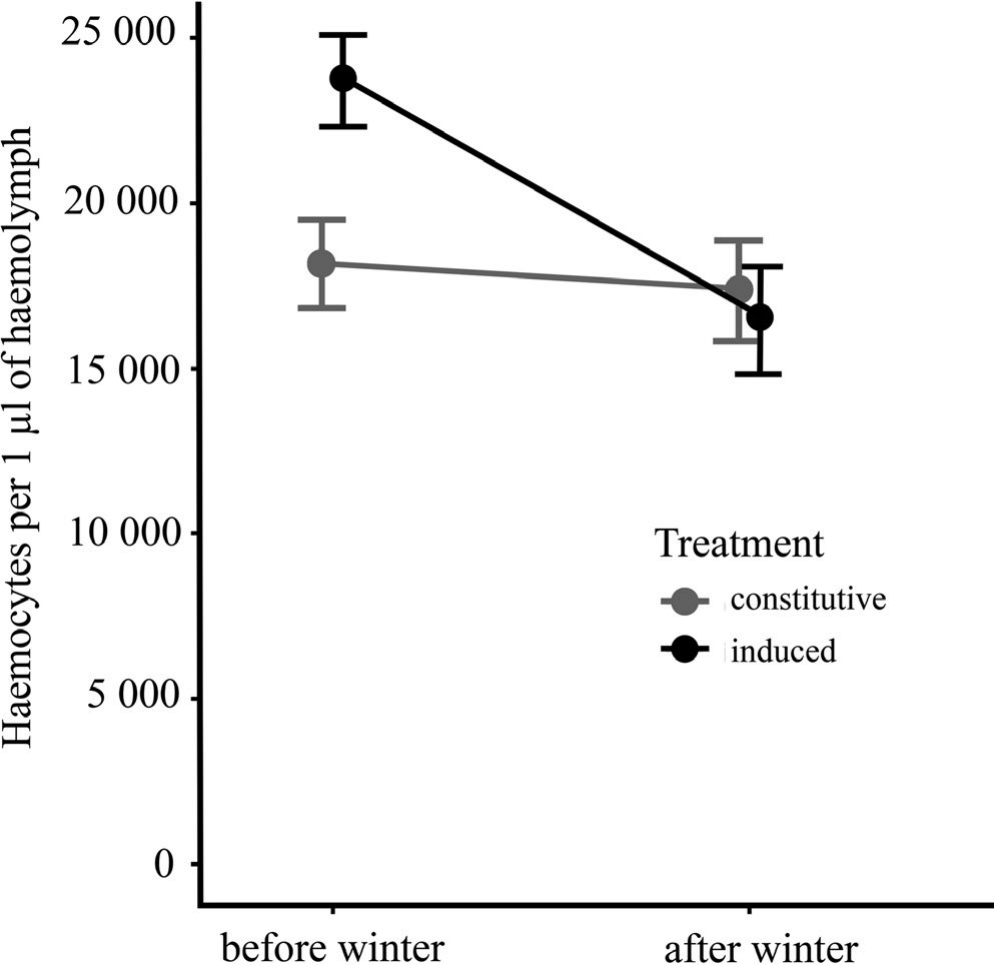
Effects of overwintering and immune challenge on total haemocyte concentration in the field‐collected *Harmonia axyridis*. Change in haemocyte concentration from autumn to spring is presented separately for naïve ladybirds (gray line) and beetles challenged by *Escherichia coli* injection 24 hours prior to measurement (black line). Means ± SEM are shown

Data for field‐collected ladybirds were consistent with the main finding obtained for laboratory‐reared ladybirds, that is, haemocyte concentrations decreased significantly from autumn to spring also for field‐collected beetles (LME: F_1,224_ = 16.96, P < 0.001; Figure 2). There was no difference in haemocyte concentration between females and males (LME: F_1,224_ = 0.05, P = 0.82) and was unaffected by immune challenge by *E. coli* (LME: F_1,224_ = 2.67, P = 0.010). However, haemocyte concentration was affected by a significant interaction between overwintering and immune challenge (LME: F_1,224_ = 9.09, P = 0.003). Haemocyte concentration was higher in challenged individuals before overwintering but the difference between challenged and naïve (untreated) individuals disappeared by the end of overwintering (Figure 2). There was also a significant interaction between sex and overwintering (LME: F_1,224_ = 7.13, P = 0.008), indicating that decrease in haemocyte concentration during winter was steeper in males compared to females.

**FIGURE 3:**
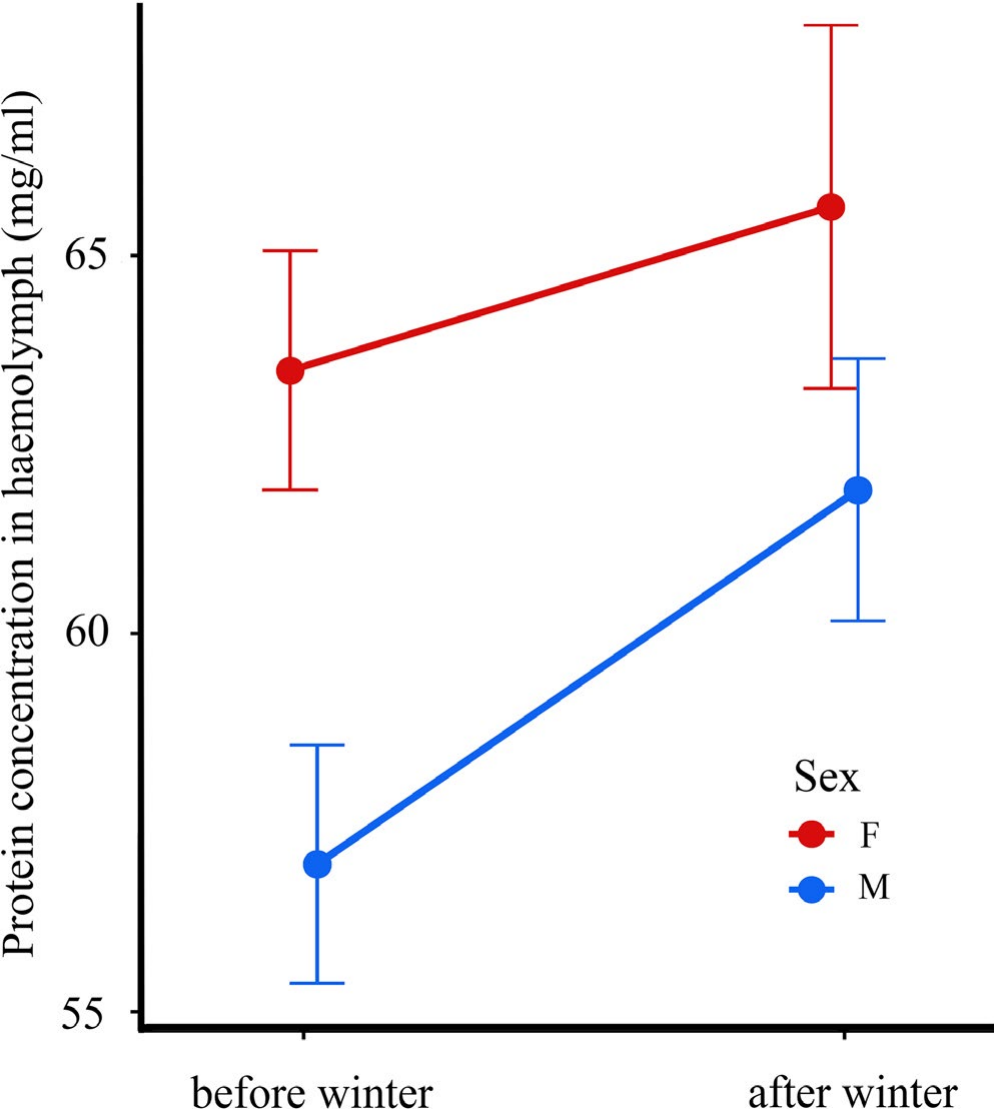
Sex‐specific effects of overwintering on total protein concentration in the field‐collected *Harmonia axyridis* ladybirds. Change in protein concentration from autumn to spring is presented separately for females (red line) and males (blue line). Means ± SEM are shown

Total protein concentration in the hemolymph of *H. axyridis* increased significantly during overwintering, that is, from late autumn to early spring (LME: F_1,224_ = 4.31, P = 0.039; Figure 3). Females had a significantly higher total protein concentration in their hemolymph compared to males (LME: F_1,224_ = 8.75, P = 0.003; Figure 3). Total protein concentration was unaffected by immune challenge (LME: F_1,224_ = 0.22, P = 0.643), and there were no significant interactions between the investigated main terms (LME: all F_1,224_ < 3.19, all P > 0.075).

**FIGURE 4:**
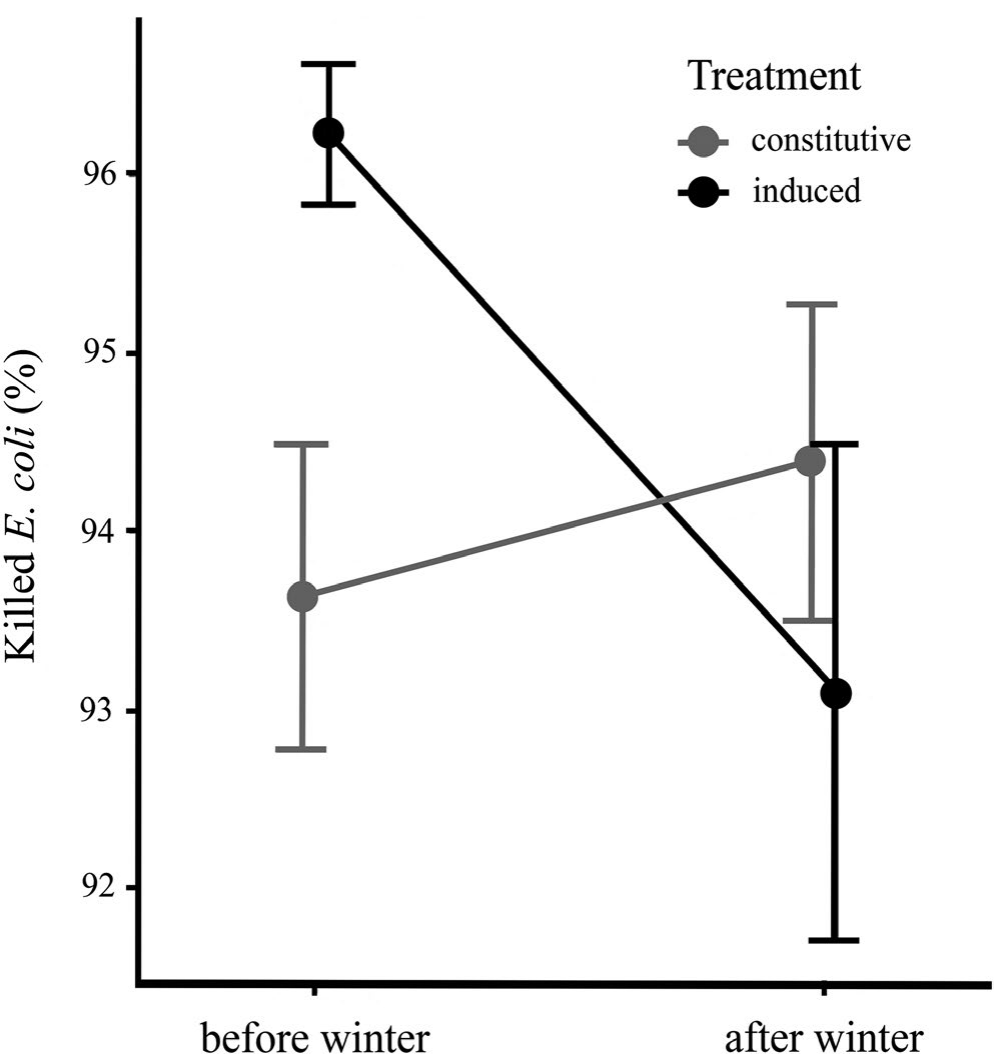
Effects of overwintering and immune challenge on antimicrobial activity against *Escherichia coli* in the field‐collected *Harmonia axyridis*. Change in antimicrobial activity against *E. coli* from autumn to spring is presented separately for naïve ladybirds (gray line) and beetles challenged by *E. coli* injection 24 hours prior to measurement (black line). Means ± SEM are shown

Antimicrobial activity against *E. coli* was not affected by any of the investigated main terms: overwintering (LME: F_1,220_ = 0,78, P = 0.38), immune challenge (LME: F_1,220_ = 2.67, P = 0.11) and sex (LME: F_1,220_ = 0.03, P = 0.86). However, there was a significant interaction between overwintering and immune challenge (LME: F_1,220_ = 6.24, P = 0.013). The immune challenge increased antimicrobial activity of *H. axyridis* hemolymph before overwintering but not after overwintering (Figure 4). Other interactions between the main terms were not significant (LME: all F < 1.94, all P > 0.16).

Interestingly, all investigated hemolymph parameters (total protein concentration, antimicrobial activity against *E. coli,* and haemocyte concentration) were significantly positively correlated within individuals and especially the relationship between total protein concentration and antimicrobial activity against *E. coli* was tight (Spearman correlation test: r = 0.44, P < 0.001; Supporting Information Figure S1).

## 4 DISCUSSION

In the present study, we investigated the effects of overwintering on selected physiological and immune parameters in *H. axyridis*, a species often used as a model for invasive insects. We revealed significant changes in haemocyte concentration in the course of overwintering for laboratory‐reared animals. However, these changes were not strongly affected by temperature regime experienced by particular beetles. For field‐collected beetles, we revealed interesting interactions between overwintering and effects of immune challenge for haemocyte concentration and antimicrobial activity against *E. coli*. These main findings indicate that *H. axyridis* may be more vulnerable to pathogens at the end of overwintering period compared to the start of overwintering period.

Changes in haemocyte concentration during overwintering found in different insect species vary from decreases (this study; Urbański et al. 2017) to increases (Ferguson and Sinclair 2017). Decreasing haemocyte concentration in the course of winter has been observed for the burying beetle *Nicrophorus vespilloides* (Urbański et al. 2017). haemocyte concentrations increased with ongoing winter in a *Curculio sp*. larvae, *Eurosta solidaginis* larvae, and adults of *Gryllus veletis*, and did not change in *Pyrrharctia isabella* larvae (Ferguson and Sinclair 2017, Ferguson et al. 2018b). The wide variation of responses indicates the presence of species‐, condition‐, or environment‐specific factors that moderate the immune response to overwintering. Unfortunately, a low number of experimental studies investigating the effects of winter period on immune responses do not allow us to make generalizations that lead to particular patterns. As temperate insects are facing a limited energy budget during overwintering (Ferguson et al. 2018a, Knapp and Řeřicha 2020), decreasing haemocyte concentrations through the winter could be linked to the high energetic costs of cellular immunity maintenance compared to humoral immunity (Ferguson et al. 2016). Note that stress in the form of limited energy sources caused by starvation, results in a reduced haemocyte concentration also during growing season in *H. axyridis* (Knapp and Řeřicha 2020). An interpretation of changes in the immune system parameters during winter is complicated by the fact that insects during overwintering have to integrate physiological responses to multiple stressors, for example, low temperature, desiccation, pathogens, and limited energy reserves, that can interact with each other (Ferguson et al. 2016, Ferguson and Sinclair 2017).

Theoretically, temperature experienced during overwintering should strongly affect immune function and long‐term adaptations to experienced thermal environment are expected (Ferguson et al. 2018a). Temperature per se modifies the activity of immune system via thermal dependence of all biochemical reactions (Sinclair et al. 2016). Moreover, effects of thermal acclimatization/acclimation on the insect immune system have been reported (Ferguson et al. 2016, Urbański et al. 2017), and stressful temperatures can both induce or dampen the activity of immune system (Marshall and Sinclair 2011, McKinstry et al. 2017, Chen et al. 2019). Surprisingly, our study revealed only a limited effect of overwintering temperature regime on haemocyte concentration. Our results indicate that haemocyte concentration decreased at only a slightly slower rate under the optimal (average) temperature regime compared to the extreme (cold) temperature regime in *H. axyridis*. However, note that the effect of the interaction between temperature and sampling period was small compared to the effect of sampling period (ongoing overwintering). Comparable studies investigating parameters of the insect immune system across multiple winter temperature regimes are scarce. To our knowledge, the only study published by Vesterlund et al. (2014) found no direct effect of overwintering temperature on phenoloxidase activity in *Bombus lucorum*; however, the phenoloxidase activity was affected by body size at warmer winter temperature, while this relationship was missing at lower winter temperature. Therefore, future studies investigating the effects of winter temperature on a wide range of immune parameters (note that responses of cellular and humoral parts of immune system to temperature can differ; Murdock et al. 2012) for a wide range of insect species are needed to improve our ability to predict the effects of ongoing climate change on functioning of insect immune system during winter.

Our data gathered for field‐collected ladybirds indicate that both cellular and humoral components of the *H. axyridis* immune system were only slightly affected by ongoing overwintering in naïve beetles, but there were significant differences in hemolymph parameters between pre‐ and postoverwintering periods in *E. coli* challenged beetles. A similar trend of reduced induced immune response (encapsulation rate) at the end of overwintering period has also been reported in males of the water strider *Aquarius najas* (Krams et al. 2011). Under optimal conditions, the immune system of *H. axyridis* is inducible and immune‐challenged individuals can, for example, show an increased ability to kill *E. coli* bacteria (Gross et al. 2010). This pattern can be explained by increased expression of genes responsible for the production of antimicrobial peptides following an immune challenge (Schmidtberg et al. 2013). As induced immune response increases energy expenditure (Adamo et al. 2016), a possible explanation for the low inducibility of the immune system after overwintering is limited energy budget at this stage (the energetic reserves of *H. axyridis* are very low at the end of overwintering period; Knapp and Řeřicha 2020; M. Řeřicha unpublished data). An adaptation of insect immune system to lower levels or activity of pathogens at the end of winter can be an alternative or complementary explanation. As the insect‐pathogen interactions are species‐specific and can be strongly affected by environmental conditions, the existence of such adaptations can explain various patterns observed for different insect species (Ferguson et al. 2018a). Unfortunately, comparable experimental studies are largely missing for insects.

Total protein concentration in hemolymph of *H. axyridis* slightly increased in the course of overwintering. Kubrak et al. (2014) found increased total protein levels in *Drosophila melanogaster* hemolymph few weeks after diapause termination and analogous pattern can be relevant also for other insects. Despite the production of immune‐related proteins, for example, AMPs, can be reduced at the end of overwintering, as indicated by lower antimicrobial activity levels in challenged individuals in this study, proteins with nonimmune functions can be upregulated. Proteins related to cryoprotection (Hahn and Denlinger 2007) can be accumulated during winter, and proteins with metabolic or reproductive functions can be upregulated in the early spring (Xiao et al. 2016). Interestingly, the increase in total protein concentration during winter was comparable between sexes and production of sex‐specific proteins (e.g., vitellogenin; Xiao et al. 2016, Kunc et al. 2019) was not clearly manifested in our study. Positive correlation between total protein concentration and antimicrobial activity against *E. coli* or total haemocyte concentration at the individual level can indicate that high protein concentration can be a proxy of good individual physiological condition and immune system functioning.

In conclusion, our study provides novel evidence that the ability of insect immune system to respond to immune challenge is lowered at the end of overwintering period. The temperature regime experienced during winter had only limited effect on observed patterns, but note that our temperature regimes mimicked real temperatures in upper soil level in Central Europe, and more extreme setting of temperature regimes can result in stronger effects on insect immune system. The comparison of our results with a few published studies investigating effects of overwintering on insect immune system indicates that the effects of winter on insect immune system are species‐specific, and future studies investigating physiological and immune parameters for a wider range of insect species are needed to allow make generalizations.

## CONFLICT OF INTEREST

Authors declare no conflict of interest.

## AUTHOR CONTRIBUTION

**Michal Řeřicha:** Conceptualization (equal); Data curation (lead); Formal analysis (equal); Investigation (lead); Writing‐original draft (equal); Writing‐review & editing (equal). **Pavel Dobeš:** Data curation (equal); Investigation (supporting); Methodology (equal); Resources (equal); Writing‐review & editing (equal). **Michal Knapp:** Conceptualization (equal); Data curation (equal); Formal analysis (equal); Funding acquisition (lead); Methodology (equal); Project administration (equal); Resources (equal); Writing‐original draft (equal); Writing‐review & editing (equal).

## 5 ETHICS STATEMENT

This research was carried out in accordance with the valid legislation of the Czech Republic and the European Union. No specific permits were required to perform experiments included in this study.

## Supporting information

Fig S1

Table S1

Table S2

Table S3

Table S4

## Data Availability

Raw data are available in Supplementary files (Tables [Link], [Link], [Link], [Link]) and at Mendeley Data (https://doi.org/10.17632/wrdf26ypmh.1).
