# Supplementary material for: Changes in haemolymph parameters and insect ability to respond to immune challenge during overwintering
Source: Ecol Evol. 2021 Mar 11;11(9):4267–75. doi: 10.1002/ece3.7323 (PMC8093749; doi:10.1002/ece3.7323)

Changes in haemolymph parameters and insect ability to respond to immune challenge during overwintering

MICHAL ŘEŘICHA, PAVEL DOBEŠ, MICHAL KNAPP

Figure S1

Correlation matrix for all investigated haemolymph parameters (total protein concentration, antimicrobial activity against *Escherichia coli* and total haemocyte concentration) measured during the field experiment investigating effects of overwintering on *Harmonia axyridis* immune system. Results of Spearman correlation tests are shown for relevant pairs of variables.

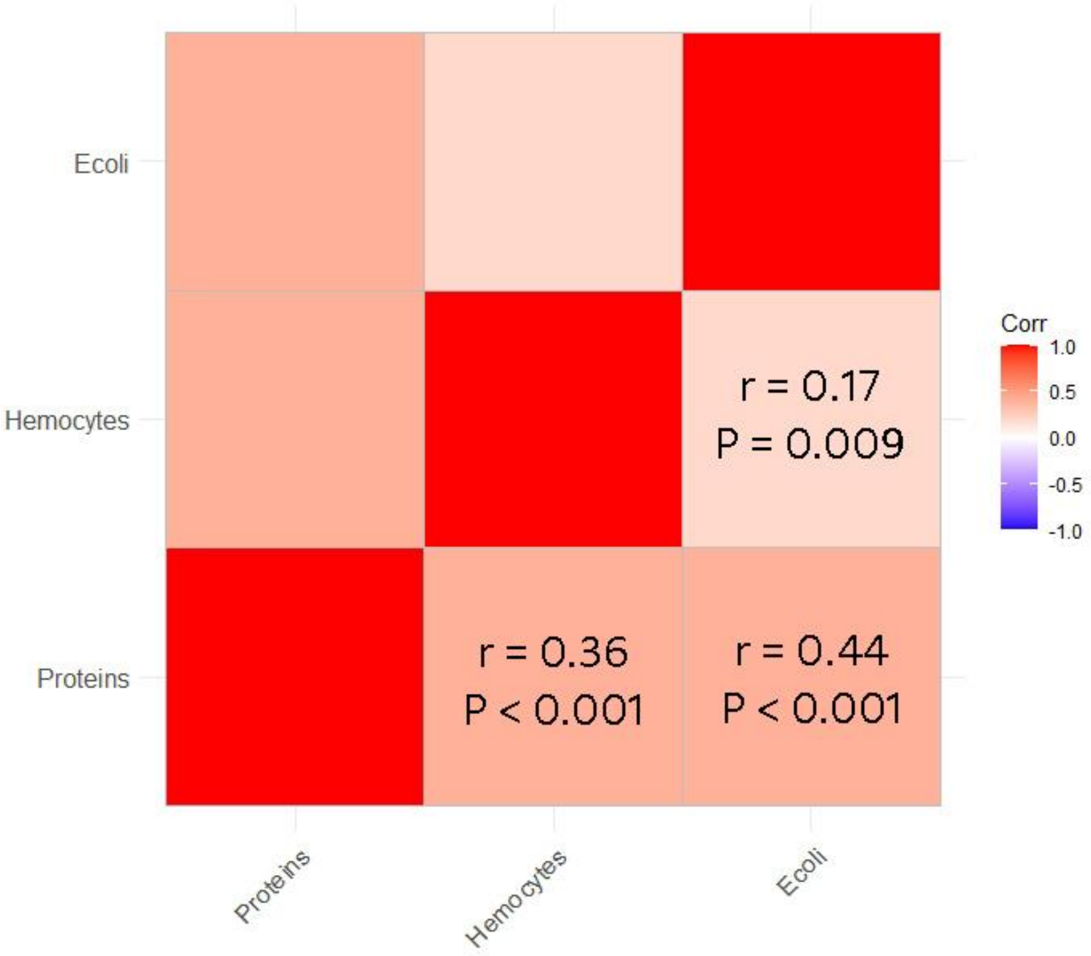

Supplement: Supplementary file 1 — Fig S1 [file ECE3-11-4267-s002.pdf]
